# A Field Study Implementing New Monitoring Technology for Roof Caving and Systematic Monitoring for Gob-Side Entry Retaining via Roof Cutting in Underground Coal Mining

**DOI:** 10.3390/s23073555

**Published:** 2023-03-28

**Authors:** Ying Chen, Zikai Zhang, Chen Cao, Zhaoju Zhang, Jun Han, Qianjia Hui, Bingjie Huo, Fengshuo Jia, Zhijie Zhu, Yang Chen

**Affiliations:** 1 Mining Engineering School, Liaoning Technical University, Fuxin 123000, China; 2 Research Center for Rock Burst Control, Shandong Energy Group Co., Ltd., Jinan 250014, China

**Keywords:** longwall mining, roof cutting, roadway retaining, goaf pressure monitoring, dynamic optimization, field study

## Abstract

The longwall mining method with gob-side entry retaining via roof cutting is a new underground coal mining method which has the characteristics of a high resource recovery ratio and environmental friendliness. Due to the complexity of this method, the research method of case-based dynamic on-site monitoring, analysis, adjustment, and optimization is usually adopted. Based on a roadway retaining via roof cutting project, in addition to the traditional indirect monitoring method of hydraulic support pressure, this study innovatively establishes a direct monitoring method for roof caving by monitoring the gangue pressure in the goaf, which provides data for the roof cutting effect and offers a new method for studying the overlying strata movement. In the project, a comprehensive monitoring and analysis system was established, including gangue pressure, cable bolt stress, bracket pressure, roadway deformation, and roof separation, which was used to dynamically analyze the effect of roof cutting and optimize the support design. The results show that the pressure of the hydraulic support close to the roof cutting is low, indicating that roof cutting is favorable in the roadway retaining mining method. The roadway deformation in the advanced abutment pressure area of the working face is small. The mining-induced stress caused by the collapse and compaction of the overlying strata in the goaf is the dominant factor affecting the effect of roadway retaining, especially in the 50–100 m range behind the working face, where the dynamic load causes high bearing capacity of the support elements, large roadway convergence, and roof separation. Temporary support and supplementary reinforcement should be added when necessary. The monitoring system presented in this study is highly comprehensive, simple, reliable, and low in cost, providing a reference for roof cutting roadway retaining projects and roof caving-related studies.

## 1. Introduction

**Figure 1:**
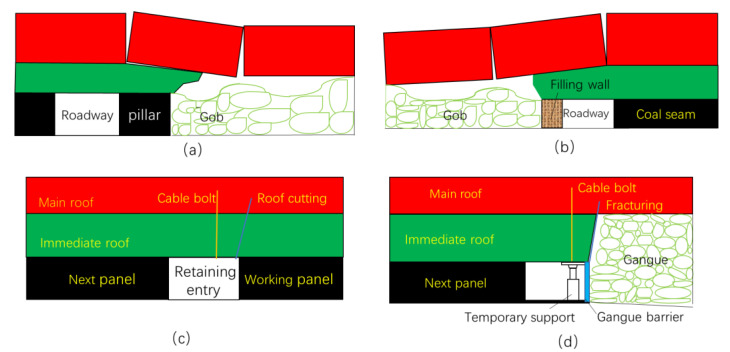
Longwall mining methods: (**a**) double entry; (**b**) gob-side entry retaining via filling; (**c**) entry retaining via roof cutting before mining; and (**d**) entry retaining via roof cutting after mining.

Coal is an important resource. Coal mining faces the challenges of sustainable development, such as improving resource utilization and reducing its environmental impacts. The longwall mining method with double entry is a traditional underground coal mining method and is the most widely used method at present [1,2]. However, the resource recovery ratio of the double entry mining method is less than 50%, mainly caused by the remaining coal pillar (generally 20–50 m, Figure 1a). Not only does the remaining coal pillar waste natural resources, but it also causes uneven subsidence of the overlying strata, damages underground aquifers, leads to ground fractures, and prevents land reuse in long-term.

From the 1980s, artificial coal pillars began to be used in some coal mines, replacing the coal pillar to improve the recovery ratio, which is called the gob-side entry retaining via filling mining method [3,4]. At present, cement slurry mixed with gangue is often used as the material to build the artificial pillar with a width of 2–5 m, as shown in Figure 1b. The filling method improves the recovery ratio of the resource; however, the construction of artificial pillars is labor-intensive, requires a large amount of materials, and is high in cost [5,6,7,8,9,10,11].

In recent years, with the aim of achieving the sustainable development of underground coal mining and the further reduction in the carbon emissions caused by mining, a mining method of roadway retaining via roof cutting has been tested and applied in some coal mines [12,13,14,15]. The key step in this method is constructing a retaining wall using U-steel pre-mining so that the gangue forms the goaf side wall of the entry after mining. To eliminate the lateral force caused by roof caving, the roof between the entry and the gob is often pre-fractured via blasting, hydraulic fracturing, or dense drilling holes. The cross-sections of the entry before and after this kind of mining method are shown in Figure 1c,d.

For the longwall mining method, whether there is a coal pillar or not, the roadway in front of and behind the working face is affected by the mining pressure, namely the so-called mining pressure, which is the leading factor of roadway deformation near the working face. Because roof caving is periodic, this mining pressure before and after work is also called periodic weighting. In front of the working face, the roadway suffers the bearing pressure generated by the suspended roof behind the working face. At the rear of the working face, the load of the supporting structure is related to the length of the suspended roof and the distribution state of cracks and changes sharply with the collapse of the roof. At the same time, it also bears the impact load caused by the collapse of the roof [16,17].

Gob-side entry retaining with roof cutting is a complex project [18,19]. The roof cutting and roadway retaining operations progress at the same time around the working face, and both areas are affected by the mining-induced pressure. Ahead of the working face, roof cutting and the installation of supplementary reinforcements such as cable bolts and gangue retaining U-steel are required. Coal is mined and transported away from the working face. Behind the working face, a temporary support is added according to the mining-induced pressure and roof caving impact to maintain the stability of the retaining roadway. At the same time, the construction scheme ahead of the working face is adjusted and optimized by monitoring and observing the retained roadway behind the working face, including adjusting and optimizing the roof cutting design by monitoring the roof caving process, optimizing the reinforcement design by monitoring the load borne by the support elements, predicting the fate of the retaining roadway by monitoring roadway deformation, and preventing disasters induced by stress concentration.

Due to the great difference in geo-conditions, mining conditions, and the complexity of the process of the mining method, roadway retaining with roof cutting projects are studied case-by-case in the field. That is, the construction and monitoring are carried out at the same time, and the operation scheme is continuously adjusted and optimized based on the monitoring results until it is satisfactory. Therefore, the successfulness of a roadway retaining with roof cutting project relies on an excellent monitoring system and related data analysis method.

It is worth noting that the environment in underground coal mines is harsh, and there are strict explosion-proof requirements for underground equipment, meaning that the range of monitoring equipment available is limited and the requirements are high. At present, pressure and displacement sensors are commonly used.

Based on a roadway retaining with roof cutting project, this study established a comprehensive monitoring system for detecting key information relevant to the operation, including roof caving status, support element health, and roadway deformation. The operation can then be adjusted and optimized by analyzing the monitored results to ensure mining safety and economy. In roof caving monitoring, in addition to the traditional hydraulic support pressure analysis, an innovative goaf gangue pressure monitoring method has been developed, which provides a basis for roof cutting design and new technology in roof caving research. The joint monitoring method of cable bolt stress and roof separation is adopted to prevent roof collapse and other roof accidents. The monitoring system proposed in this study is comprehensive, highly reliable, uses simple equipment, and is low in cost. This work provides a reference for other roadway retaining with roof cutting projects.

## 2. Materials and Methods

The buried depth of the studied working face is 610.0–737.2 m, the length of the working face is 1490 m, and the width is 273 m. A total of 6 faults were exposed during the excavation, with a maximum drop of about 2 m. The overall geological structure is simple. According to the drilling data and the actually revealed geological condition during the roadway excavation, the roof of the working face is composed of fine-grained sandstone and siltstone, which are in conformity with each other. The floor is composed of siltstone and fine-grained sandstone in conformity contact.

According to the geological drilling, there is about 15 m of thick fine sandstone in the roof within 50 m of the roof at the side of the reserved roadway. During the process of retaining the roadway, the pre-fracturing cutting and support parameters should be adjusted in time, according to the actual roof conditions.

### 2.1. Gob Floor Pressure Monitoring

The roadway retaining with roof cutting mining method has the advantages of a high resource recovery ratio and environmental friendliness; however, it faces challenges related to the stability of the roadway. The key is whether the roof caves quickly and fully, which is in turn closely related to whether the roof cutting is successful. At present, the direct monitoring method for roof caving and the subsidence of the overlying strata is still in the developmental stage. A borehole drilled from ground can successfully obtain the subsidence data of the overlying strata; however, the monitoring cost is very high [20,21,22].

Currently, there is no direct monitoring method for the roof caving process underground. Microseismic monitoring has been considered as a most effective tool to understand the overlying strata movement process [23,24,25]. However, the challenges here mainly include the development of new technologies in the accuracy of sensors, the optimization of the sensor networks, the algorithms for parameter calculation, and the seismic assessment methodologies. Meanwhile, the microseismic data cannot reflect the compaction process of the gangue in the goaf. Therefore, the effect of the roof cutting operation can only be evaluated, resulting in the roof cutting design completely relying on engineering experience [26,27].

This work adopted the innovative method of deploying pressure sensors in the goaf, and the caving state of the overlying strata can be estimated according to the pressure on the floor. This method is simple, reliable, and the measurement results are intuitive. It is new for roof caving monitoring and can also be used in engineering sites with exposed goafs. However, as coal pillars or artificial pillars isolate the goaf area, this technology cannot be applied in such cases.

According to the drilling data and the revealed geological condition during the roadway excavation, the roof of the working face is composed of fine-grained sandstone and siltstone, which are in conformity with each other. The floor is composed of siltstone and fine-grained sandstone in conformity contact. According to the geological drilling, there is about 15 m of fine sandstone in the roof within 50 m of the overlaying strata. During the process of roadway retaining, the pre-fracturing cutting and support parameters should be adjusted according to the actual roof conditions.

**Figure 2:**
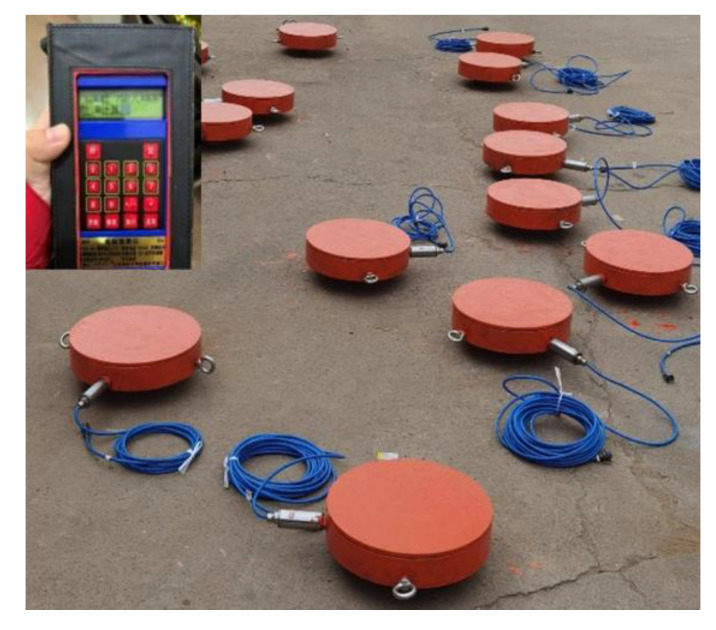
Gob gangue pressure sensor and supporting data facility.

Monitoring points for the floor pressure of the gangues were arranged in the gob. The buried depth of the roadway was 530 m. The measured vertical ground stress was 12.09 MPa. The range of the buried gangue pressure sensors was selected as 15 MPa. The customized pressure sensors buried in the goaf and the data collection facility are shown in Figure 2.

**Figure 3:**
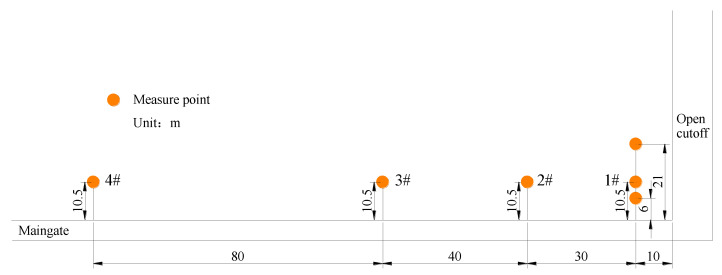
Arrangement of the gob floor pressure monitoring.

The arrangement of gangue pressure monitoring is shown in Figure 3. The first monitoring station, composed of three pressure sensors, was installed 10 m from the cutoff. The depth from the rib was 6 m, 10.5 m, and 21 m, respectively. In the following pressure measurement, one pressure sensor was arranged at an interval of 40 m. The frequency of data acquisition was daily.

### 2.2. Hydraulic Support Pressure Monitoring

The roof caving in longwall mining is periodic. The length of the overhanging roof behind the working face increases during the retreat of the working face, resulting in an increase in the hydraulic support pressure and the deformation of the roadway in front of the working face. When the roof extends to a certain length, it suddenly collapses in a large area, causing a sudden change in the stress state of the surrounding rock around the working face. This kind of mining-induced pressure change, namely dynamic load, is particularly noticeable in the range of about 40 m around the working face.

**Figure 4:**
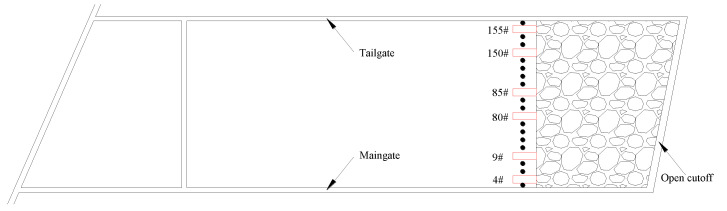
Arrangement of the working face shield pressure monitoring.

To analyze the overlying strata movement and characterize the ground pressure of the retaining roadway, the hydraulic shield pressure was monitored through the electrohydraulic controller. There were 156 10MN shields, including 3 end shields, 1 transition shield and 152 middle shields. The interval between shields was 1.75 m. We selected shields 4# and 9# near the retaining roadway side, shields 80# and 85# in the middle of the working face, and shields 150# and 155# near the tailgate as monitoring stations (Figure 4), and the dynamic pressure at the working face was analyzed according to the initial and end hydraulic pressures.

### 2.3. Monitoring of Cable Load and Roadway Deformation

**Figure 5:**
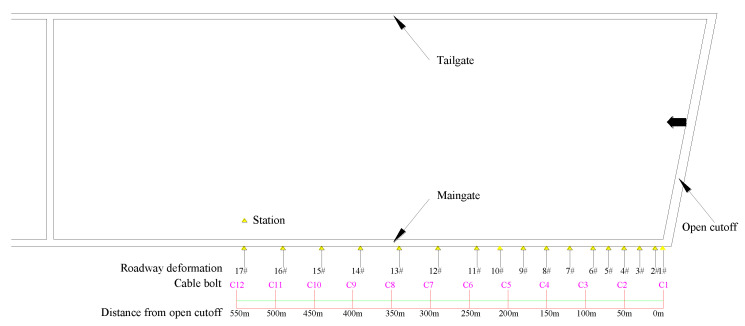
Layout of the cable axial load and roadway deformation monitoring.

The loading state monitoring of the cable bolt is an in situ detecting method for the healthy state of the support element. The main purpose is to provide a basis for adjusting the parameters of the support system. A cable dynamometer was used for cable axial load monitoring. It consisted of a pressure box with a pressure gauge. During installation, the cable was stressed, and the pressure was recorded, and then it was read regularly to obtain the axial load of the cable. The axial load of the cable was monitored in the retaining roadway, and the interval between each monitoring station was 50 m, as shown in Figure 5.

The retaining entry is used for the next working face; therefore, it has certain dimensional requirements for the roadway cross section. The retaining roadway is more difficult to repair than pillar roadways. If the section cannot meet the production needs of the next panel, the retaining entry project is considered to have failed. Therefore, it is necessary to monitor the roadway convergence to ensure the availability of the roadway within the entire service life. At the same time, roof separation is the main cause of roof collapse accidents, so roof subsidence and separation monitoring are the key measures to prevent roof collapse accidents.

In this study, the tunnel convergence was monitored by means of crossing measurement at each monitoring station. The automatic online equipment of a roof separation sensor was used to collect real-time data of roof separation. The monitoring station was deployed as shown in Figure 5.

It is worth noting that the service life of the roadway is often 2–3 years; therefore, the support design principles are safety and cost savings. Therefore, it is common for the surrounding rock support element in local areas to yield or even fail. However, the working state of support elements in the ground’s civil engineering is usually designed to be within the elastic deformational range, and it is quite different between them.

### 2.4. Pressure Monitoring of Prop

In a periodic weighting circle, although the bolts, props, U-steel, etc., in the roadway have the same structure, their loading state may be quite different due to different roof states behind the working face. Therefore, the health monitoring and maintenance of support elements guarantee roadway stability.

**Figure 6:**
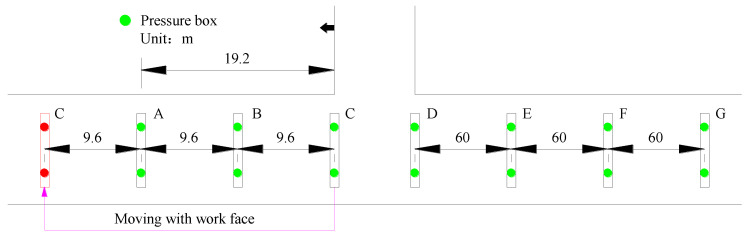
Monitoring the deployment of hydraulic props.

Pressure monitoring was carried out for the props before and after the working face. The bearing pressure of the props ahead of the working face changed sharply due to abutment pressure. Therefore, pressure boxes were arranged at an interval of 9.6 m within 20 m ahead of the working face. There were three stations in total, with two measuring points and one rib in each station. The data were read manually once per day (Figure 6).

The hydraulic prop behind the working face acted as a kind of temporary support, mainly used to prevent the impact load generated by roof caving and maintain the stability of the roadway in the process of gangue compaction. Therefore, pressure monitoring was arranged at an interval of 60 m within 180 m behind the working face. There were two sensors in each group, with one rib each. The acquisition frequency was once every two days.

## 3. Results

### 3.1. Results and Analysis of Gangue Pressure

**Figure 7:**
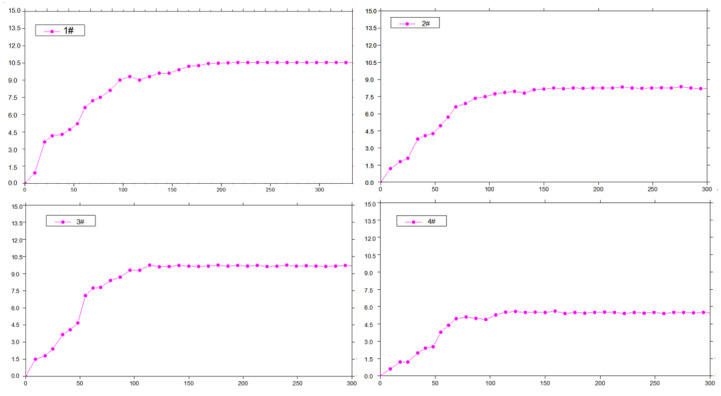
The measured floor pressure. The *x*−axis is the distance from the working face in meters, and the *y*−axis is the floor pressure in MPa.

Gangue pressure monitoring points were arranged in the goaf, and the data receiving end was placed in the retained roadway. Four groups of pressure measuring stations were selected for analysis. Monitoring points 1#, 2#, 3#, and 4# were 10 m, 40 m, 80 m, and 160 m away from the cutoff, respectively, as shown in Figure 3. The curve of the floor pressure of the four measurement points in the goaf is shown in Figure 7.

This curve shows that the change trend of the floor pressure in the goaf is similar. After the working face passes, the roof gradually collapses, and the pressure evidently rises at 30–80 m behind the working face, after which it increases gently. The floor pressure tends to be stable from 110 m away from the working face. This shows that the overlying strata tend to be stable 80–110 m behind the working face.

### 3.2. Result Analysis of Hydraulic Shield Pressure

**Figure 8:**
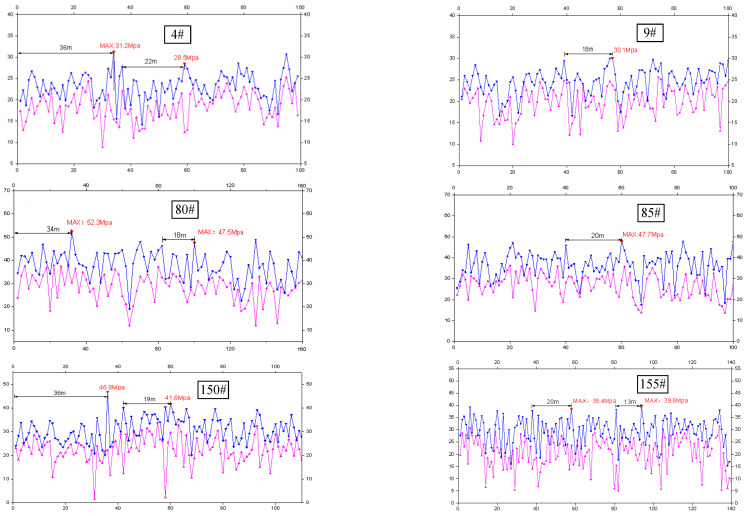
The hydraulic shield pressure. The *x*−axis is the distance from the cutoff, in meters; the *y*−axis is the pressure in MPa; the shallow line is the initial pressure; and the deep colored curve is the pressure at the end of the circle.

Six hydraulic shields (4#, 9#, 80#, 85#, 150# and 155#) were selected for abutment pressure analysis. Among them, shields 4# and 9# are close to the roof cutting, shields 80# and 85# are located in the middle of the working face; and shields 150# and 155# are located near the tailgate, far from the roof cutting operation. The working resistance of each hydraulic shield is shown in Figure 8.

The results show that the initial weighting distance near the retaining entry is 36 m and the maximum pressure is 31 MPa; its periodic weighting distance is 18–22 m, and the peak pressure is 30.1 MPa. The initial weighting distance in the middle of the working face is 34 m, with a peak pressure of 52.3 MPa; its periodic weighting distance is 17–20 m, with a peak pressure of 47.7 MPa. The initial weighting distance of the working face near the tailgate is 36 m, and the peak pressure is 46.8 MPa; and the periodic weighting distance is 13–20 m, and the peak pressure is 39.8 MPa.

This indicates that the roof cutting has less effect on the initial and periodic weighting distances, but the pressure of the hydraulic shield near the roof cutting is significantly low, indicating that the roof caving around the roof cutting is timely and more sufficient, which is consistent with the expected outcome. The pressure of the hydraulic shield in the middle of the working face is the largest. This may be caused by the backward convex curve of the roof caving layout [28,29]. It is proposed that the bearing pressure in the middle of the working face is the key to the weighting distance.

### 3.3. Analysis of Cable Stress and Roadway Deformation

**Figure 9:**
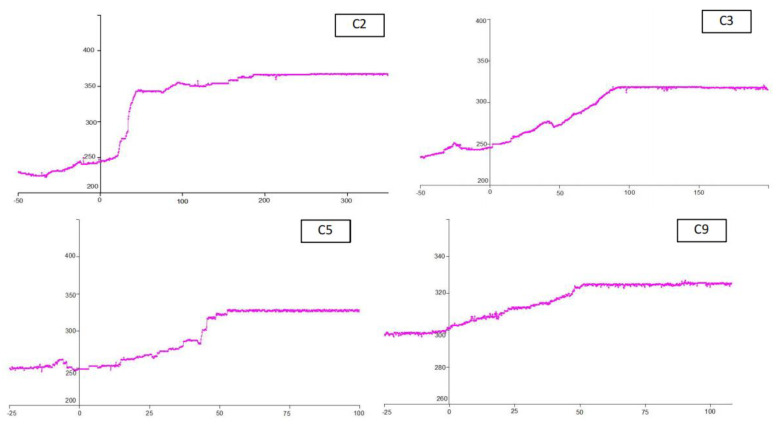
Cable bolt axial load curves. The *x*−axis is the distance from the working face, in meters; the *y*−axis is the load in kN.

We selected C2, C3, C5, and C9 measuring points as typical monitoring results for analysis. The measuring points are located 50 m, 100 m, 200 m, and 400 m away from the open cutoff, respectively. The monitoring results are shown in Figure 9.

The loading characteristics of cables can be obtained through curve analysis. Table 1 shows the statistics of the positions and peak stresses of each cable at the selected measuring station.

The results show that the abutment pressure ahead of the working face has little influence on the load of the cable bolt. The stress of the cable increases slightly within the range of 12–22 m ahead of the working face. The main reason is that the roof is relatively hard, and the abutment pressure has less influence on the roadway deformation.

In the retained entry, the pressure of the cable increases significantly. The peak tension of the C2 cable is 366 kN, which is close to the yielding load. The load increases mainly occurred 43~61 m behind the working face, which indicates that the main roof subsidence played a role in the cable bearing. The area for temporary support and reinforcement is about 50 m behind the working face.

The cable load curve shows that there are two modes for load increment of the cable—one is a slow increase behind the working face, such as for C9, while the other is a sudden increase, such as at the C2 measuring point. The reason for this is related to the position of the roof fracture line under periodic weighting. The former cable is located between two periodic weighting fracture lines, and its pressure increases slowly with the increase in the size of the suspended roof. The latter cable is affected by weighting and roof caving, and its stress increases instantly with roof fracturing.

**Figure 10:**
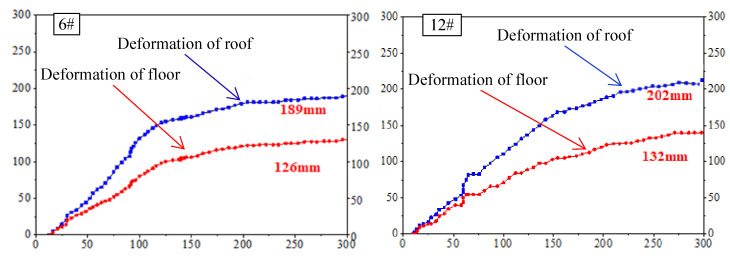
Deformation of the retained entry. The *x*−axis is the distance behind the working face, in meters; the *y*−axis is the displacement in mm.

A typical result of the retaining roadway deformation is shown in Figure 10. It shows that the surrounding rock converges continuously within 100 m behind the working face. After 130 m behind the working face, the convergence rate of the surrounding rock of the roadway tends to be gentle, and it is proposed that the gangue in the goaf tends to be compacted; meanwhile, the transition stage of gangue compaction is 100–130 m.

According to the deformation statistics at different locations of the retaining roadways, the deformation of the roadways at 150–180 m behind the working face is small, and most sections of the roadway become stable at 200 m behind the working face. Therefore, considering the surrounding rock deformation, it is suggested that the retained roadway 220 m behind the working face is in a stable state—that is, the temporary support can be withdrawn after 220 m.

**Figure 11:**
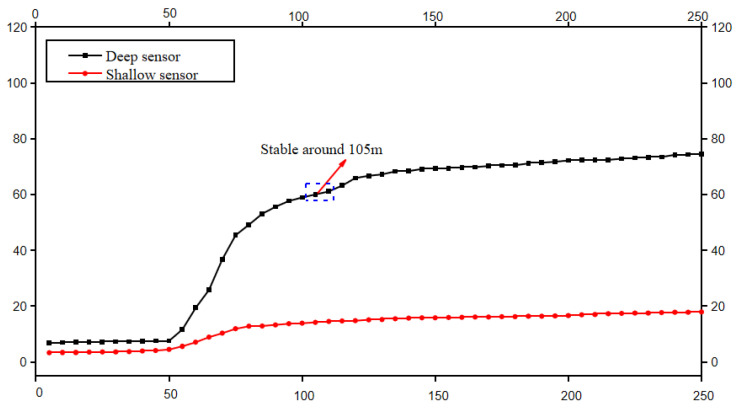
Roof separation monitoring result (station from open cutoff 300 m). The *x*−axis is the distance behind the working face, in meters; the *y*−axis is the displacement in mm.

The monitoring results of roof separation are similar to the results of the surrounding rock deformation. The monitoring results of a typical roof separation are shown in Figure 11. The result shows that the roof separation mainly occurs 50–100 m behind the working face, representing the collapse and subsidence of the overlying strata in the goaf. The displacement of the separation is generally less than 80 mm and tends to be stable 150 m behind the working face. It is considered that the probability of a roof collapse is low.

### 3.4. Analysis of Prop Pressure

**Figure 12:**
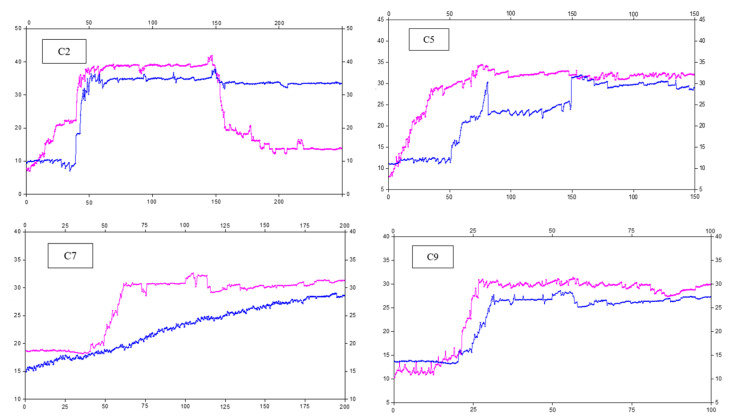
Pressure curve of the props, blue curve is on the coal rib, pink curve is on the gob rib. The *x*−axis is the distance behind the working face, in meters; the *y*−axis is the pressure in MPa.

The pressures of the C2, C5, C7, and C9 stations are selected as typical results for analysis. Their positions are 50 m, 200 m, 300 m, and 400 m away from the open cutoff, respectively. The pressure curves of the props are shown in Figure 12.

Statistically, it is found that the pressure of the prop near the roof cutting is relatively large, which corresponds to the large roof subsidence near the roof cutting. The monomer pressure gradually increases at 20–50 m behind the working face. The pressure of prop C7 increases rapidly from 50 m behind the working face. Beyond that, some monomer pressures fluctuate, while others increase slowly. In addition, some monomer pressures are large, causing pressure relief.

The pressure development mode of the prop is complex. The pressure of prop C9 suddenly increases to 31 MPa when it is 24 m away from the working face and then fluctuates. The pressure of prop C5 increases slowly to a stable state. It is believed that the monomer pressure behind the working face has a relationship with the geological conditions, roof deformational state, and roof cutting effect described in this section. Different reinforcement support methods should be considered based on the actual situation to achieve the best support effect for the retaining roadway.

## 4. Conclusions

The mining method of the gob-side entry retaining with roof cutting mining method has the characteristics of a high resource recovery ratio and environmental friendliness. However, this method has complex technological needs and is studied case by case via on-site monitoring, analysis, adjustment, and optimization. Based on a roadway retaining with roof cutting project, this study systematically monitored the gangue pressure, hydraulic shield pressure, roadway deformation, roof separation, cable axial stress, and prop pressure. Conclusions can be drawn as follows:

- A direct monitoring method of gangue pressure in the goaf is proposed in this study—it is simple, reliable, and highly innovative. The monitoring data can be used to evaluate the effect of roof cutting. In this project, the monitoring data show that the collapse and compaction of overlying strata in the goaf mainly occurred 30–80 m behind the working face.
- The pressure analysis results of the hydraulic shield show that the support pressure at the roof cutting is noticeably low, which is conducive to maintaining the stability of the roadway.
- The monitoring of anchor cable stress and surrounding rock deformation shows that the deformation of the roadway is small in front of the working face. The mining-induced stress generated by the collapse and compaction of the overlying strata in the goaf is the main factor affecting the effect of roadway retaining.
- The bearing capacity of support elements 60–120 m behind the working face is high. Along with large roadway convergence and roof separation, temporary support and reinforcement should be considered when necessary.
- The monitoring system proposed in this study is comprehensive in content, high in reliability, uses simple equipment, and is low in cost, providing a reference for roadway retaining projects and for other overlying strata caving studies.

## Figures and Tables

**Table 1:** Positions and peak tensile stresses of selected cables.

Cable	Distance from Open Cutoff/m	Load Noticeable Increase (Distance from Working Face/m)	Peak Axial Load/kN
C2	50	47	366.5
C3	100	61	318.3
C5	200	43	326.8
C9	400	50	325.3

## Data Availability

The data used to support the findings of this study are available from the corresponding author upon reasonable request (chenying@lntu.edu.cn).
